# Lateral unicompartmental knee arthroplasty through a medial parapatellar approach: surgical technique and mid-term results in 108 patients

**DOI:** 10.1007/s00402-026-06274-8

**Published:** 2026-03-30

**Authors:** Filippo Leggieri, Lorenzo Braconi, Mattia Chirico, Paolo Salari, Andrea Baldini

**Affiliations:** 1 Department of Clinical Orthopaedics, University of Florence, Florence, Italy; 2 Istituto per Artroprotesi Complesse e Revisioni, Casa di Cura Villa Ulivella, Firenze, Italy

**Keywords:** Lateral unicompartmental knee arthroplasty, Medial parapatellar approach, Surgical technique, Implant survivorship, Patient-reported outcomes, Satisfaction

## Abstract

**Introduction:**

Lateral unicompartmental knee arthroplasty (L-UKA) is typically performed through a lateral parapatellar approach. Few studies of L-UKA performed through a medial approach are published. We aimed to present surgical tips, and mid-term clinical results and survivorships of L-UKA performed through a medial approach.

**Methods:**

We retrospectively reviewed single-centre single-surgeon data from L-UKA using medial parapatellar approach between 2016 and 2023. Patients with follow-up < 12 months were excluded. Primary endpoint was implant survivorship (time from index surgery to revision or last follow-up). Secondary endpoints included the rates of patients achieving Patient Acceptable Symptom State (PASS) thresholds: ≥ 67.5 for Knee Society Score-Knee (KSS-K) and ≥ 70.5 for Knee Society Score-Function (KSS-F). Survival analysis was used for implant survivorship. Logistic regression was performed for factors potentially associated with failure to achieve the PASS thresholds. Significance was set at *P* < 0.05.

**Results:**

Among 110 patients initially identified, two died before 12-month follow-up and were excluded from clinical outcome analysis, leaving 108 patients for functional assessment at a mean 47.6 months ± 24.5 of follow-up (range 12–96.7 months). No complications were reported during hospitalization. There were four failures (3.7%) overall: two cases (1.8%) of OA progression, one (0.9%) of aseptic loosening, and one case (0.9%) of periprosthetic fracture of the medial condyle requiring revision surgery. All 110 patients were included in survival analysis, with the 2 patients who died before 12 months included as censored data at time of death. The overall 7-year survivorship was 97.2% (95% CI 94.2–100%), declining to 92.1% at 84 months when the last revision occurred. PASS thresholds were achieved by 99.0% patients for KSS-Knee and by 86.1% for the KSS-F. Patient age was a significant predictor of failure to achieve functional PASS thresholds (OR = 1.3 per year, 95% CI 1.0–1.6, *p* = 0.036).

**Conclusions:**

L-UKA performed through a medial parapatellar approach yielded excellent clinical outcomes and survivorship at mid-term follow-up.

**Level of evidence:**

IV (retrospective case series).

## Introduction

Approximately 10% of patients with severe knee osteoarthritis (OA) present with an isolated disease of the lateral compartment [1–3]. Unicompartmental knee arthroplasty (UKA) represents only 7.1% of all knee arthroplasty procedures [4], with lateral UKAs (L-UKA) accounting for merely 1% [5, 6]. L-UKAs offer several advantages over TKA in patients with isolated compartmental disease, including lower complication rates, faster recovery, reduced morbidity, and greater cost-effectiveness [7–9]. However, L-UKAs remain underutilized, primarily due to their reported higher revision rates and technical challenges compared to TKAs. Current evidence demonstrated a correlation between low surgical volume and increased failure rates in medial and L-UKA procedures [10–12].

L-UKAs present additional technical challenges compared to their medial counterparts because the reduced surgical lateral window mobility makes surgical exposure more difficult. Consequently, the lateral approach—which displaces the patella medially—has been traditionally preferred for surgical exposure [13, 14]. The lateral approach dominates current practice. To date, only three reports of L-UKA performed through a medial approach have been published, all characterized by very limited case numbers and lacking clinically meaningful outcome metrics or detailed surgical technique descriptions [15–18].

Therefore, we presented the mid-term outcomes from a cohort of L-UKAs performed using a medial parapatellar approach and provided a detailed step-by-step description of the surgical technique. Our hypothesis was that the medial approach may offer advantages in terms of surgical familiarity and reproducibility, without compromising implant survival and clinical outcomes.

## Materials and methods

### Design and patient population

We conducted a retrospective investigation of a consecutive series of primary L-UKAs performed at a single institution between October 2016 and November 2023 using the Zimmer Unicompartmental Knee (ZUK, Enovis Corporation, Delaware, U.S.). All procedures were performed by a single fellowship-trained surgeon using the medial parapatellar approach. The study adhered to the principles outlined in the Declaration of Helsinki. The study was conducted according to the STROBE guidelines [19].

**Fig. 1:**
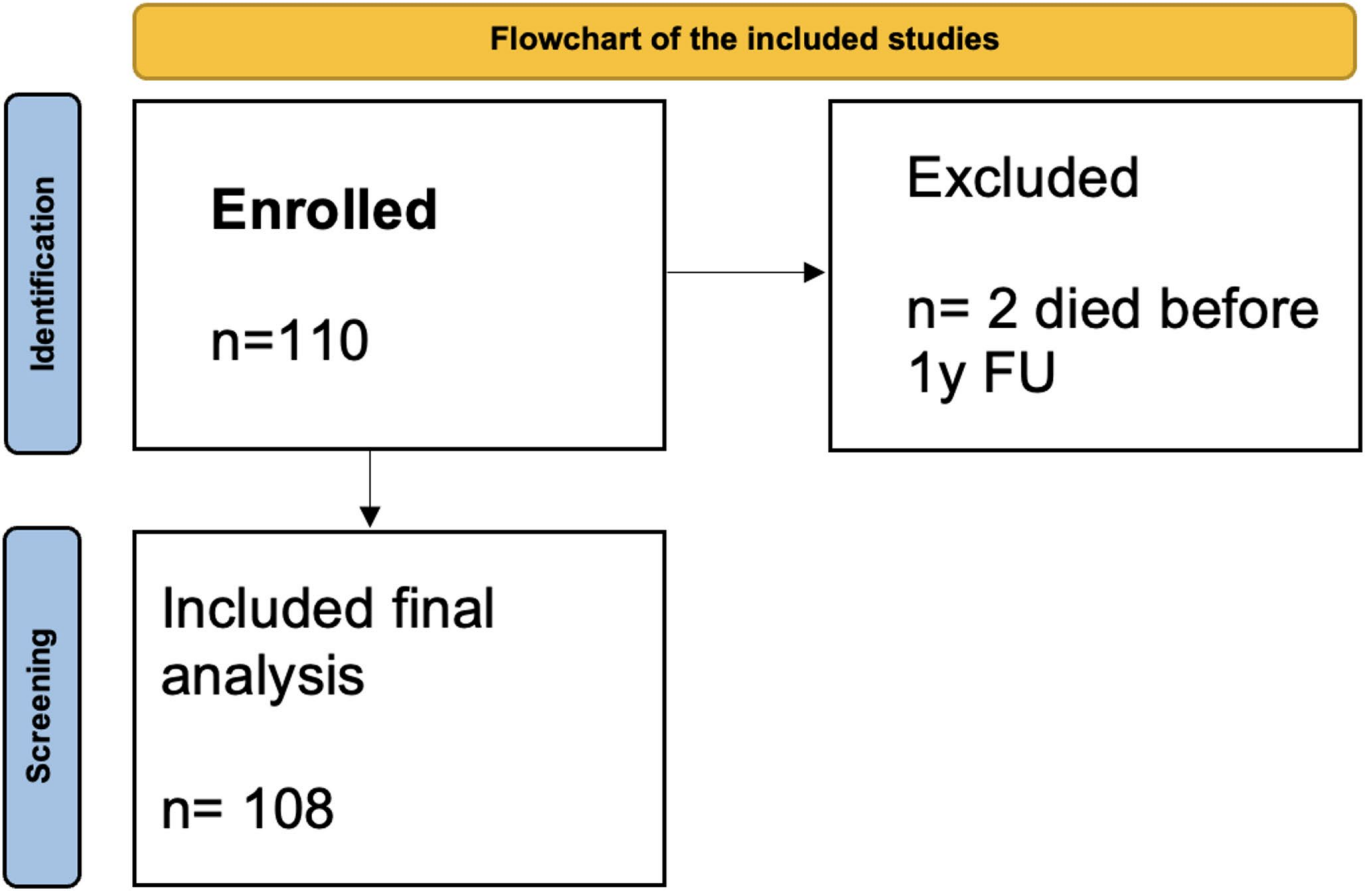
Flowchart of the included population. Two patients who died before 12-month follow-up were excluded from functional outcome analysis but included in survival analysis as censored data

**Fig. 2:**
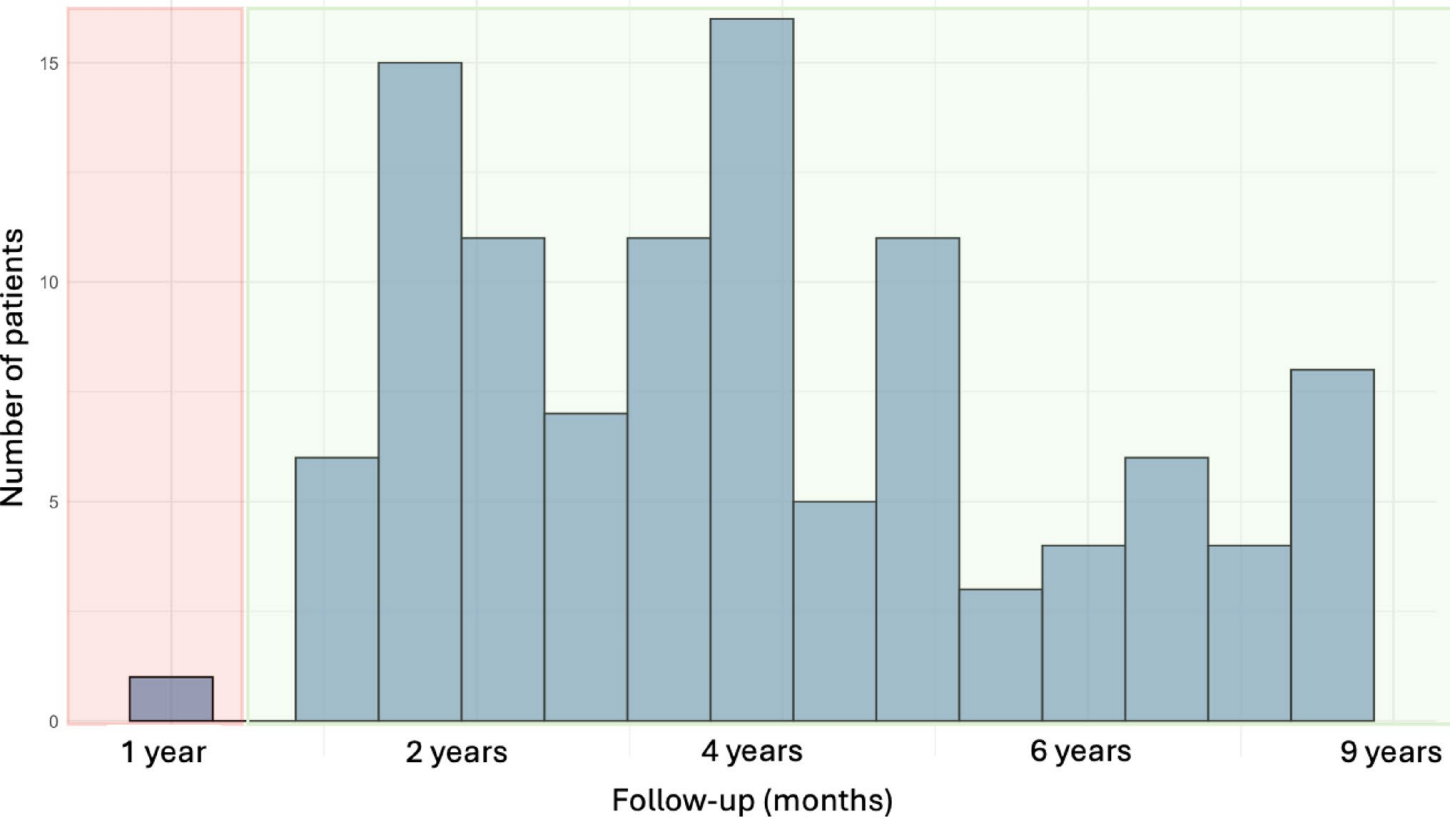
Follow-up distribution. The highlighted area in red shows patients who were not included in the data analysis, while the highlighted area in green the patients who were included in the analysis

All patients who underwent unilateral or one-stage bilateral primary L-UKA using the medial parapatellar approach were included. For functional outcome analysis, cases with less than 12 months of follow-up were excluded. Two patients died before reaching 12-month follow-up and were therefore excluded from functional outcome analysis, though they were included in the survival analysis as censored data at their time of death. No patients were lost to follow-up or withdrew consent. After exclusions, 108 patients were available for functional outcome analysis (Fig. 1), predominantly female (*n* = 71, 66.6%) with a mean age of 68.6 ± 11.3 years (range 49–89 years, Fig. 2).

### Data collection

Baseline demographics were collected, including age, gender, side, ethnicity, body mass index, and surgery date. Clinical parameters included OA grade according to Kellgren-Lawrence classification (KL) [16], presence of patellofemoral OA, primary or post-traumatic OA etiology, osteonecrosis, use of tourniquet, surgery time, and complication rates including revision, periprosthetic joint infection (PJI) [20], periprosthetic fracture, or any additional procedures involving the affected knee. Between December 2024 and January 2025, patients were evaluated during scheduled outpatient clinical assessments to obtain follow-up data and Knee Society Score measurements [21].

###  Endpoints and outcome measures

The primary endpoint was implant survivorship, defined as time from index surgery to revision of any component or last follow-up without revision. Patients who died before implant failure were censored at time of death. Patients lost to follow-up were censored at their last known contact date. The secondary endpoint evaluated the rates of patients achieving favourable clinically meaningful outcomes defined by Patient Acceptable Symptom State (PASS) thresholds for postoperative Knee Society Score results, and the identification of predictors associated with failure to achieve PASS for KSS-Function using logistic regression analysis. PASS thresholds were defined as a Knee Society Score-Knee (KSS-K) ≥ 67.5 and a Knee Society Score-Function (KSS-F) ≥ 70.5 [22]. For functional outcome analysis, patients were required to have minimum 12-month follow-up.

### Preoperative surgical protocol

All surgical procedures were performed through midline skin incision with a medial limited parapatellar arthrotomy. A pneumatic tourniquet, when utilized, was inflated at 250 mmHg during exposure and bone preparation, was deflated to check for the inferolateral genicular artery bleeding and reinflated for the cementation phase. All patients received standardized perioperative antibiotic prophylaxis consisting of Cefazolin 2g IV, and a multimodal protocol for bleeding and pain control including tranexamic acid, dexamethasone, cryocompression and low opioid usage.

### Surgical technique

#### Patient positioning and surgical approach

The patient was positioned on the operating table with a single distal roll bar, allowing the affected knee to flex to approximately 90°. A lateral support was positioned at the proximal thigh, directly over the tourniquet or its virtual position.

The procedure began with a standard median parapatellar arthrotomy through an approximately 10 cm incision slightly medial to the midline. During the medial arthrotomy, care was taken to preserve the anterior horn of the medial meniscus. Optimal compartment exposure was achieved by placing a Langenbeck retractor medially and a bent Hohmann retractor laterally. Patella was displaced laterally without eversion and the distal part of the arthrotomy was completed taking care not to incise the anterior horn of the medial meniscus. Fat pad resection was limited to what is required for adequate visualization of the lateral tibial resection. No ligament releases were usually required. The lateral meniscus was removed to allow adequate exposure of the lateral tibial plateau.

Proper exposure of the knee requires maintaining the bent Hohmann retractor laterally to keep the patella laterally displaced, while the Langenbeck retractor is positioned medial to retract skin that may obstruct the surgeon’s visualization. Lateral patella facectectomy was performed in extension with partial patellar eversion.

#### Tibial resection

To optimize exposure and access to the lateral compartment during tibial preparation, the assistant kept the limb in a “figure-of-four” position with the foot internally rotated. This position facilitated opening in flexion of the lateral compartment,

The tibial cut was performed first with an extramedullary guide, with the goal of reestablishing neutral coronal alignment to the mechanical axis of the tibia and a minimal slope of 2–3°. Overall alignment target was around 2–3° of residual hip-knee-ankle (HKA) angle. It is important to note that the minimum thickness 8 mm polyethylene insert typically reduces the hip-knee-ankle (HKA) angle by approximately 3° in an average height patient when performing a pure resurfacing procedure. The amount of tibial resection and the final polyethylene thickness ultimately determined the final HKA alignment.

The initial sagittal cut was made using a reciprocating saw immediately adjacent and lateral to the cruciate ligaments with the aim of internally rotating approximately 10° the resection by pointing to the mid-to-medial third of the tibial tuberosity. The transverse final cut is then completed using a 15 mm wide oscillating saw blade.

Removal of the lateral osteotomized plateau could be difficult due to the peripheral osteophytes rim. For this purpose, a bent Hohmann retractor was placed to act as a lever on the femoral condyle, pushing the tibial fragment outward over an osteotome positioned along the tibial osteotomy line.

#### Femoral resection

For the femoral cuts, the knee was positioned at 90° of flexion, with the same bent Hohmann placed laterally and the Langenbeck retractor medially to retract the skin. If the joint space remained insufficient to position the guide, the figure-of-four position could also be utilized.

Femoral cutting guide was positioned as much as possible lateral on the condyle profile, and 2 mm posteriorized (either using posterior shims or bone wax) to compensate for the built-in posterior femoral over-resection included in the ZUK system which was originally designed for the anteromedial osteoarthritis.

Both femoral and tibial components were cemented using low-viscosity Simplex bone cement (Stryker, Kalamazoo, Michigan, U.S.)

### Postoperative protocol

All patients followed the same postoperative care protocol, including a rapid recovery standardized pain management and rehabilitation program according to our institutional practice. Deep vein thrombosis (DVT) prophylaxis consisted of early mobilization after surgery and daily low molecular weight heparin (LMWH) injections for 21 days.

### Data analysis

Data distribution was analysed using means, medians, ranges, and interquartile ranges for continuous variables and using frequencies and percentages for categorical variables. Normality of continuous variables was assessed using the Shapiro-Wilk test. All continuous variables showed non-normal distributions. Implant survivorship was evaluated using Kaplan-Meier survival analysis with 95% confidence intervals (CI). Patients who were lost to follow-up and those who died before the final follow-up without implant failure were included as censored data in the survival analysis. Survival analysis was performed on a per-knee basis, with each knee each knee treated as an independent observation. To analyse factors potentially associated with failure to achieve the PASS threshold for KSS-Function, an exploratory multivariable logistic regression analysis was performed. Variables included in the model were age, follow-up duration, surgery time, and tourniquet time. Variables for multivariable analysis were selected based on clinical relevance and potential influence on functional outcomes. Gender, BMI, and K-L grade were not included as preliminary univariate analysis showed no significant associations (*p* > 0.10 for all), and to avoid model overfitting given the limited number of events (*n* = 15 patients not achieving PASS). All statistical analyses were performed using R software version 4.1.0 (R Foundation for Statistical Computing, Vienna, Austria). Statistical significance was set at *P* < 0.05. Missing data across all variables were less than 5% of the total dataset. Little’s test for missing completely at random was performed, confirming that data were missing at random (*P* = 0.736).

## Results

A total of 110 patients were initially identified for the study. Among these, two patients died before the first 12 months of follow-up and were excluded. One patient who underwent two-stage bilateral L-UKA died at 45.45 and 23.52 months from the first and second procedures respectively and was included as censored data. The mean final follow-up for the included 108 patients was 47.6 months ± 24.5 (range 12–96.7 months). Patients’ characteristics are presented in Table 1.. No complications were reported during hospitalization.

**Table 1.** Patient's characteristics

Variable	Mean	Median	SD	Min	Q1	Q3	Max
Tourniquet time	26.1	27	7.2	12	20	31.5	37
Surgery time	51.2	50	12.1	35	45	60	85
Final KSSKnee	93.2	95	7	60	90	100	100
Final KSSFunction	88.7	90	13.1	50	80	100	100
Patello femoral OA [n(%)]	4	(3.7)					
Osteonecrosis [n(%)]	2	(1.8)					
Primary OA [n(%)]	104	(96.3)					
Post-traumatic OA [n(%)]	8	(7.4)					
KLgrade 1 [n(%)]	1	(0.9)					
KLgrade 2 [n(%)]	3	(2.7)					
KLgrade 3 [n(%)]	29	(26.8)					
KLgrade 4 [n(%)]	75	(69.4)					
PFJ [n(%)]	4	(3.7)					
Without tourniquet [n(%)]	71	(65.7)					

Primary OA refers to osteoarthritis of the lateral compartment. Patellofemoral OA indicates concomitant patellofemoral involvement in addition to lateral compartment disease.

*KL* kellgren-lawrence, *PFJ* patellofemoral joint, *OA* osteoarthritis, *KSS* knee society score, *SD* standard deviation, *Min* minimum, *Q1* first quartile, *Q3* third quartile, *Max* maximum

There were four failures (3.7%), of which two occurred in one patient who underwent bilateral two-stage L-UKA with OA progression on both knees. Overall, there were two cases (1.8%) of OA progression, one (0.9%) of aseptic loosening, and one case (0.9%) of periprosthetic fracture of the medial condyle requiring revision surgery.

**Fig. 3:**
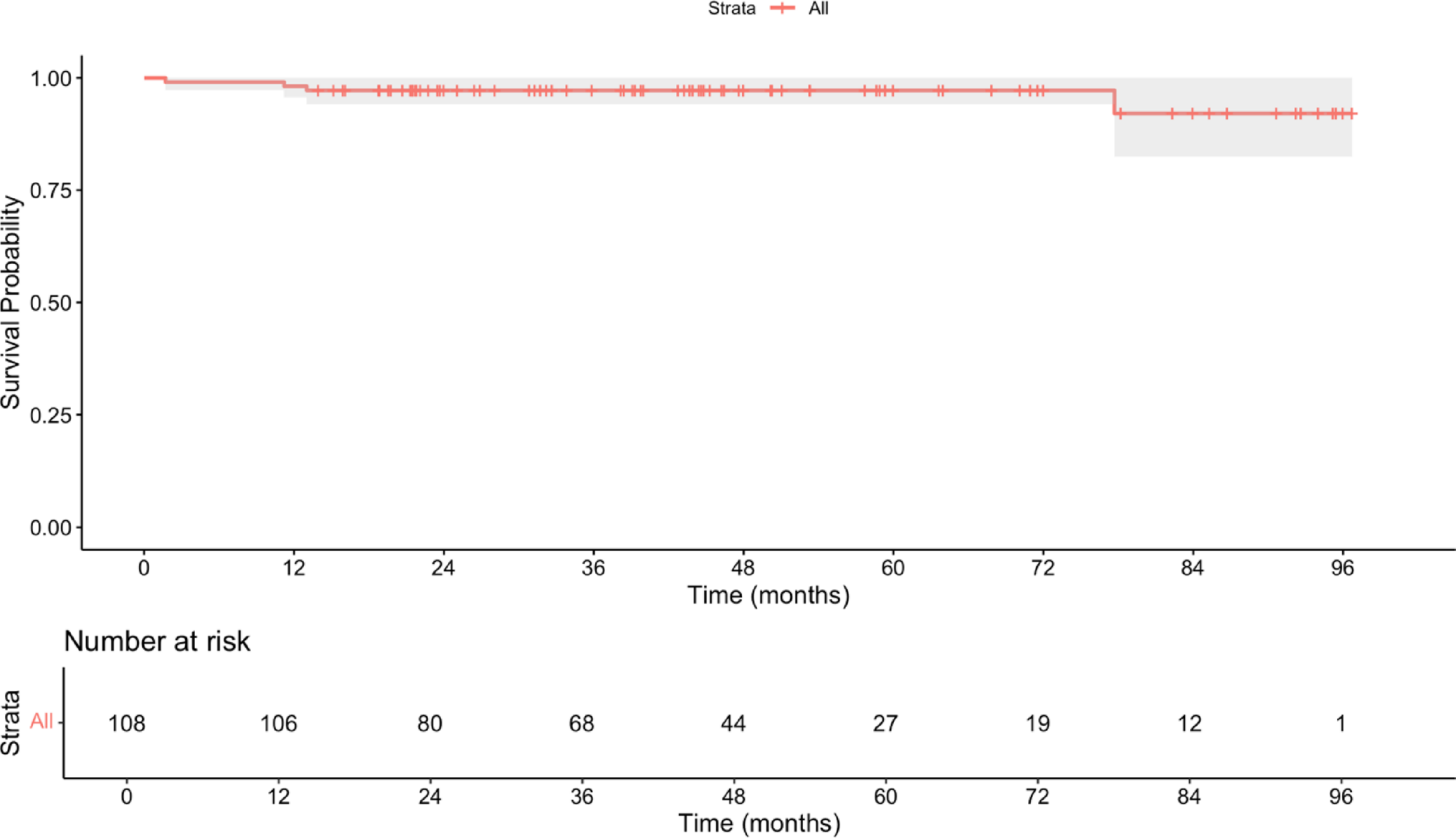
Kaplan-Meier survival curve

The mean survival time for L-UKA was 94.3 months (SE = 1.4 months, 95% CI 91.5–97.0, Fig. 3). The survivorship remained stable at 97.2% through 72 months, with one late revision occurring at 84 months, reducing survivorship to 92.1% at 7 years. Beyond 84 months, only 1 patient remained at risk, limiting interpretability of longer-term estimates. The median survival time was not reached during the study period, indicating that more than 50% of the implants remained functional at the longest follow-up. Table 2 presents the detailed survival analysis showing the number of knees at risk, events, and survival probabilities at each time point. The Patient Acceptable Symptom State (PASS) threshold was achieved by 99.0% of patients (107/108) for the KSS Knee score (≥ 67.5) and 86.1% (93/108) for the KSS Function score (≥ 70.5). Overall, 86.1% of patients achieved acceptable symptom states for both knee and function domains.

**Table 2:** Kaplan-Meier Survival Analysis

Time (months)	Number at risk	Number of events	Survival probability	Standard error	Lower 95% CI	Upper 95% CI
0	108	0	1.000	0.0	1.000	1.000
12−1 year	106	2	0.981	0.01	0.956	1.000
24	80	1	0.972	0.01	0.942	1.000
36	68	0	0.972	0.01	0.942	1.000
48−3 years	44	0	0.972	0.01	0.942	1.000
60−5 years	27	0	0.972	0.01	0.942	1.000
72	19	0	0.972	0.01	0.942	1.000
84	12	1	0.921	0.05	0.825	1.000
96−8 years	1	0	0.921	0.05	0.825	1.000

*CI *confidence interval

To investigate the lower rate of patients achieving PASS for KSS-F, the logistic regression model identified age as the only significant predictor (Table 3). Each one-year increase in age was associated with a 30% increase in the odds of not achieving PASS for function, which was typical in the octogenarian cohort of the study group (OR = 1.3, 95% CI 1.0–1.6.0.6, *p* = 0.036).

**Table 3:** Logistic regression evaluating potential predictors for not achieving PASS for KSS-F

	Beta	S.E.	Sig.	OR	95% CI	
					Lower	Upper
Age	0.2	0.1	0.036	1.3	1.0	1.6
Surgery Time	-0.04	0.1	0.643	1.0	0.8	1.1
Follow-up	0.02	0.03	0.544	1.0	1.0	1.1
Tourniquet Time	0.01	0.1	0.957	1.0	0.8	1.2
*PASS* patient acceptable symptom state, *KSS-F* knee society score-function, *S.E*. standard error, *Sig.* significance, *OR* odds ratio, *CI* confidence interval						

## Discussion

The main finding of the current study was that L-UKA performed through a medial parapatellar approach demonstrated satisfactory clinical outcomes and survivorship at mid-term follow-up. Our series of 108 L-UKAs revealed a survivorship of 97.2% maintained through 6 years, declining to 92.1% at 7 years (84 months) after one late revision. While survival data extended to 96 months, the number of patients at risk beyond 84 months was limited; therefore, we report survivorship up to 7 years as the most reliable estimate.

Nearly all patients achieved knee-specific satisfaction thresholds, while the vast majority also reached functional satisfaction benchmarks, indicating high overall levels of patient satisfaction with the procedure. The logistic regression analysis revealed that age was the only significant predictor of failing to achieve functional PASS thresholds, explaining why fewer patients achieved functional satisfaction compared to knee-specific satisfaction, likely reflecting age-related declines in baseline mobility, muscle strength, and activity levels rather than any surgical or implant-related deficiency.

This study addressed a gap in the surgical management of lateral compartment OA by providing the largest published series of L-UKA performed through a medial parapatellar approach, with detailed surgical technique descriptions and mid-term outcomes. This is particularly relevant given the established correlation between low surgical volume and increased failure rates in UKA procedures [11, 12, 23], as a potentially more accessible and reproducible technique may help surgeons maintain adequate proficiency even with lower case volumes. Despite the technical challenges conventionally associated with L-UKA exposure, none of the failures were directly attributable to the surgical approach itself and did not suggest any approach-specific complications.

The 2024 Australian Arthroplasty Registry Report showed cumulative percent revision rates for primary unicompartmental knee replacements (with primary diagnosis of osteoarthritis) of 1.9% at 1 year, 4.4% at 3 years, 6.1% at 5 years, and 11.3% at 10 years [24]. Our revision rate of 3.7% at a mean follow-up of 47.6 months is comparable to the registry’s reported rates. Limited evidence evaluating L-UKA with medial approach exists, limiting potential comparison. Edmiston et al. reported 1 case out of 13 (7%) requiring revision of UKA with medial approach at 12.6 years of follow-up, also demonstrating no difference in the revision rate between the medial approach (*n* = 1/13, 7%) and lateral approach (3/52, 5%) among L-UKAs (*P* = 1.000) [17]. Scott et al. reported no revisions and no notable soft-tissue complications in forty-eight L-UKAs performed between 1991 and 2004 at a mean follow-up of 5.2 years postoperatively [25]. Wei et al. reported excellent patient satisfaction at 1-month postoperative follow-up, up to 95% [15]. None of the three reports presented clinically meaningful metrics in their series.

While L-UKA represents a valuable treatment option offering significant advantages over total knee arthroplasty, it remains severely underutilized [14, 26, 27]. A major barrier to wider adoption is the technical challenge associated with the conventional lateral approach, which requires displacing the patella medially—a less familiar manoeuvre for most surgeons who predominantly perform medial compartment procedures. A medial arthrotomy may offer potential benefits for L-UKA procedures. The approach is familiar and recognizable to most surgeons, particularly those who perform L-UKAs less frequently. It provides potentially improved visualization of the entire knee joint, including crucial anatomical landmarks for precise implant positioning, trochlear structures, and the medial compartment, while facilitating accessory surgical gestures [28, 29]. The medial approach allows easier intraoperative conversion to patellofemoral arthroplasty or revision procedures if necessary. It has also been suggested that the medial approach may better preserve the joint capsule’s natural vascularity, which predominantly flows from medial to lateral, potentially promoting enhanced wound healing—although this has been primarily described in the context of total knee arthroplasty and direct evidence in the UKA setting is lacking [30, 31]. Finally, this approach creates more favorable scar conditions for any potential future reinterventions [32].

Particular attention must be paid to femoral component positioning to account for the typical posterolateral wear pattern in valgus knees, especially when using non-anatomical implants by repurposing a contralateral medial component [33–36]. However, this is a shared issue among valgus knees treated with non-anatomical implants. The technique we describe—including the use of shims or bone wax under the distal portion of the femoral cut guide—effectively addresses this issue by posteriorizing the guide and ensuring uniform cut thickness. This precision in femoral resurfacing directly translates to optimal soft tissue balance and maximal bone stock preservation, maintaining the natural soft tissue patterns that are critical to physiologic knee function [37, 38]. This may translate into mid-term greater functional outcomes, higher patient’s satisfaction rates, and preservation of natural knee biomechanics which is essential for high-demand activities and mid-term implant survivorship [39].

The strengths of our study include the largest sample size for a L-UKA series with mid term follow-up, the use of validated outcome measures, and the application of PASS thresholds to provide clinically meaningful interpretations of outcomes. Furthermore, all procedures were performed by a single surgeon, minimizing technical variability.

Based on our findings, L-UKA via medial approach may be considered as a viable alternative. The medial approach offers a reproducible, familiar technique that may lower technical barriers for surgeons with lower L-UKA volumes, potentially expanding appropriate utilization of this procedure.

Several limitations should be acknowledged. The retrospective design introduced potential selection bias; the lack of a direct comparison group with L-UKA performed through a lateral approach prevented definitive conclusions about the superiority of one approach over the other; The number of patients at risk beyond 84 months was limited to one, precluding reliable long-term survivorship estimates. Systematic postoperative radiographic assessment of limb alignment and component positioning was not performed, as this was not part of our institutional protocol and could not be retrospectively applied. This limits the ability to correlate radiographic parameters with clinical outcomes, which should be addressed in future prospective studies. Furthermore, all procedures were performed by a single fellowship-trained, high-volume surgeon at a single institution; outcomes achieved in this setting may not be directly generalisable to lower-volume centres or surgeons with less experience in lateral unicompartmental arthroplasty.

Future research should include direct comparative studies between lateral and medial approaches for L-UKA especially in the early term, ideally in the form of randomized controlled trials. Additionally, investigations into learning curve effects, component positioning, and long-term outcomes would further elucidate the relative merits of each approach. This approach should also be validated using robotic-assisted systems.

## Conclusions

L-UKA performed through a medial parapatellar approach demonstrated acceptable clinical outcomes and implant survivorship at mid-term follow-up of up to seven years. These findings suggest that the medial parapatellar approach is a feasible and reproducible surgical option for lateral compartment osteoarthritis. Whether this approach confers advantages over the conventional lateral approach in terms of surgical accessibility or broader adoption remains to be established through direct comparative studies.

## Data Availability

The datasets used and/or analyzed during the current study are available from the corresponding author on request.
